# Exploring Factors Associated with Quality of Life in Caregivers of Children and Adolescents with Sickle Cell Disease and HIV: A Comparative Analysis

**DOI:** 10.1155/2024/4429541

**Published:** 2024-03-06

**Authors:** Charlotte Eposse Ekoube, Dora Mbonjo Bitsie, Erero F. Njiengwe, Edgar Mandeng Ma Linwa, Christian Eyoum, Ritha Mbono Betoko, Jeannette Disso Massako, Emmanuel Heles Nsang, Abba Soumaiyatou, Callixte Tegueu Kuate

**Affiliations:** ^1^Faculty of Medicine and Pharmaceutical Sciences, University of Douala, Douala, Cameroon; ^2^Laquintinie Hospital of Douala, Douala, Cameroon; ^3^Faculty of Sciences, University of Douala, Douala, Cameroon; ^4^Faculty of Health Sciences, University of Buea, Buea, Cameroon; ^5^Faculty of Medicine and Biomedical Sciences, University of Yaounde I, Yaounde, Cameroon

## Abstract

**Introduction:**

Paediatric HIV and sickle cell disease (SCD) are two stigmatising and potentially fatal illnesses that place a significant burden on families. HIV patients benefit from a longstanding free-service national programme in Cameroon, and this could considerably alleviate burden of care on HIV caregivers, possibly leading to better quality of life (QoL) in HIV caregivers compared to SCD caregivers. Our study aimed to compare the QoL between caregivers of children and adolescents with SCD and HIV and explore factors associated with this QoL in Cameroon.

**Methods and Materials:**

We conducted a hospital-based cross-sectional analytic study at Douala Laquintinie Hospital from February to May 2023. A questionnaire was administered to caregivers of paediatric patients (≤18 years) with SCD and HIV. The Pediatrics Quality of Life-Family Impact Module (PedsQL FIM), the 7-item Generalized Anxiety Disorder (GAD-7), and the 9-item Patient Health Question (PHQ-9) tools were used as measures of quality of life, anxiety, and depression, respectively. Multivariable linear regression was used to determine factors associated with quality of life. A significance level was set at *p* < 0.05.

**Results:**

We included 199 caregivers: SCD = 104 and HIV = 95. The mean age of caregivers in our sample was 40.47 ± 10.18 years. Caregivers of paediatric patients with HIV had a better mean quality of life than SCD (93.01 ± 7.35SD versus 64.86 ± 9.20SD, *p* < 0.001). PHQ-9 score (*B* = −1.52, 95% CI = [-2.08; −0.96], *p*=<0.001), GAD-7 score (*B* = −1.46, 95% CI = [-2.09; −0.83], *p*=<0.001), spending less than 75 000 FCFA on medications monthly (*B* = 12.13, 95% CI = [5.73; 18.94], *p*=<0.001), and being a SCD caregiver (*B* = −11.62, 95% CI = [-18.46; −4.78], *p*=0.001) were factors independently associated with quality of life on multivariable analysis.

**Conclusion:**

Quality of life is lower in caregivers of children and adolescents with SCD than with HIV. Preventing depression and anxiety as well as advocating for the subsidization of medications through a national SCD program may improve quality of life in SCD caregivers.

## 1. Introduction

Sickle cell disease (SCD) is one of the most common genetic blood disorders [1]. It manifests clinically as painful vaso-occlusive crises and chronic haemolytic anemia with episodes of acute worsening and bacterial infections, causing heavy morbidity and mortality in countries with limited resources [2–4]. According to available evidence, Nigeria is believed to have the highest number of individuals affected by sickle cell disease on a global scale [5]. In Cameroon, population carrier frequencies of SCD range from 8% to 34%, with a birth incidence of 1.6% [6]. SCD is a chronic illness that impacts physical and social development [7, 8]. Similar to many other paediatric chronic illnesses, SCD places a significant burden on children and their families that may manifest as symptoms of depression and anxiety [1, 9, 10]. The prevalence of depression in caregivers of patients with SCD has been reported as high as 68.9% [1]. This impact on families is worse in low- and middle-income countries due to inadequate social welfare and healthcare services [11]. Clinical severity of SCD has been reported as a major factor influencing ability to cope [1, 6].

Paediatric human immunodeficiency virus (HIV) is a similar stigmatising, debilitating, and potentially fatal illness associated with a high burden of care. According to UNAIDS statistics, 39 million people were living with HIV globally in 2022 and 1.5 million (3.3%) of these people were children aged between 0 and 14 years [12]. In Cameroon, as of 2021, there were 510 000 people living with HIV, of which 31 000 (6.1%) were children aged 0–14 years [13]. HIV patients benefit from a longstanding free-service national programme which considerably alleviates burden of care [14]. The introduction of free highly active antiretroviral therapy (HAART) has reduced mortality and morbidity among patients living with HIV/AIDS (PLWHA) with a consequent increase in lifespan and quality of life [15, 16].

Quality of life is a multidimensional concept influenced by various factors and varies among individuals and cultures, including objective measures of well-being and subjective perceptions of happiness and fulfilment [17]. Research findings indicate that as a child's illness advances, both the child and their caregivers undergo a decline in quality of life, leading to a notable deterioration in their physical, psychological, social, and economic welfare [18]. Caregivers of children with chronic illnesses, such as sickle cell disease and HIV, may experience significant impacts on their quality of life due to increased stress, financial strain, social isolation, and changes in routines [19, 20].

Understanding the quality of life in these caregivers is crucial to identify support areas, intervene effectively, and improve the well-being of both caregivers and the children and adolescents they care for. This study specifically focuses on comparing the quality of life (QoL) of caregivers of children and adolescents with sickle cell disease and HIV and exploring factors associated with this QoL in Cameroon.

## 2. Methodology

### 2.1. Study Design and Setting

This was a hospital based cross-sectional analytic study. This design allowed direct/simultaneous comparison between the two groups of interest and also permitted efficient data collection and analysis with the limited resources available in our setting. The study was conducted at the sickle cell center and the HIV management unit, located at Douala Laquintinie Hospital (DLH), a second category health structure in Akwa, Douala, Cameroon. The sickle cell center is the only center exclusively dedicated to the care of patients with SCD in the city of Douala. The unit has nine hospitalisation rooms with a capacity of 21 beds. It is managed by a 17-man staff: two pediatricians, two general practitioners, one clinical psychologist, 11 nurses, and one secretary. The HIV management unit is a day care hospital providing medical consultations, counseling, drug dispensation, and follow-up of PLWHA.

#### 2.1.1. Study Period

The study was conducted over four months, from 1st of February to 31st of May 2023.

### 2.2. Sampling and Study Population

Convenience sampling, a nonprobability technique, was used to select participants based on their availability and accessibility, specifically targeting caregivers attending routine consultations at Laquintinie for recruitment. We assumed that many caregivers may be unwilling to participate because of the time constraints and fear of stigma.

The study population was made up of two comparison groups: caregivers of paediatric patients with SCD and HIV. We included caregivers aged 21 years and above, residing with at least one living patient with SCD or HIV aged <18 years and whose disease was confirmed by a laboratory diagnosis.

We excluded caregivers who refused to give informed consent for participation in the study or had a previously diagnosed psychiatric or psychological disease or caregivers of patients with additional chronic diseases like chronic kidney disease, cancer, or congenital heart diseases. Caregivers of patients with a cumulative diagnosis of HIV and SCD were also excluded from the study.

### 2.3. Sample Size Calculation

The primary outcome in this study is the QoL score as measured by the Paediatric Quality of Life Family Impact Module (PedsQL FIM) scale. The main objective of the study was to compare the mean quality of life scores between the two caregivers' subpopulations: HIV and SCD. No study in Africa reporting PedsQL FIM scores in caregivers of patients with HIV was found. This is why chronic kidney disease was used as a surrogate reference.

The total sample size for the study was calculated using the formula [21]:

(1) $$\begin{array}{c} N=\frac{\left(r+1\right){\left(Z_{\alpha /2}+Z_{1-\beta }\right)}^{2}\sigma^{2}}{rd^{2}}, \end{array}$$

where *Z*_*α*_ is the normal deviate at a level of significance (*Z*_*α*_ is 1.96 for 5% level of significance and 2.58 for 1% level of significance), *Z*_1−*β*_ is the normal deviate at 1-*β*% power with *β*% of type II error (0.84 at 80% power), *r* = *n*1/*n*2 is the ratio of sample size required for 2 groups, which was kept as one for keeping equal sample size for 2 groups, D is the difference of means of 2 groups, and *σ* is the pooled standard deviation of the 2 groups [√(SD1^2^+ SD2^2^)/2]. These values were obtained from Bethany et al. in Kenya [1] and Manal et al. in Egypt [22] with the mean PedsQL FIM scores of 43.76 ± 17.53 and 51.9 ± 22.2, respectively.

From this formula, the minimum sample size required was 95 participants in each group. Thus, an overall minimum sample size of 190 patients was necessary for the study to have 80% power, significance of 5%, and 95% confidence interval.

### 2.4. Data Collection Tools

A questionnaire was pretested on 10 caregivers of patients with SCD and 10 caregivers of patients with HIV to assess the challenges with the administration of the questionnaire (language, sequence, time constraint, and understanding) and adjusted accordingly. The pretested questionnaire gathered patient information, including age, gender, year of diagnosis, recent hospitalisation, and educational status, as well as caregiver information such as age, gender, ethnic group, education level, marital and working status, relationship with the patient, estimated monthly revenue, and various expenses. To minimize biases, all expenses were categorized based on multiples of the recognized minimum wages in Cameroon [23] and reference from a Nigerian study on cost of care for SCD [24]. A cutoff value of 75,000 FCFA was chosen to represent expenditures in the sample. However, the study did not measure the level of social and emotional support in a standardised manner.

### 2.5. Paediatric Quality of Life Family Impact Module (PedsQL FIM)

The Paediatric Quality of Life Family Impact Module (PedsQL FIM) scale was used to assess quality of life. It was created in 2004 to assess the impact on families and parents with medically fragile children [25]. The PedsQL FIM scale assesses the physical, emotional, social, and cognitive functioning, as well as communication and concern, as reported by the parents. Family interactions and everyday activities as described by parents are also measured by the module. Since responses to questions are based on parental perceptions of their children's quality of life (QoL), there are no cutoff values established for what is considered to be “bad” QoL for families. Reponses are on a 5-point Likert scale. Detailed description of the PedsQL FIM scale is available online [26]. In the overall sample, in SCD caregivers and HIV caregivers, the total PedsQL FIM scale had a Cronbach alpha coefficient at 0.95, 0.93, and 0.84, respectively.

Because evidence suggests that anxiety, depression, and QoL are associated, we sought to measure these concepts with standardised and validated tools [27]. We used the GAD-7 and PHQ-9 tools in this study.

### 2.6. Generalized Anxiety Disorder Scale (GAD-7)

The seven-item Generalized Anxiety Disorder scale (GAD-7) was used to diagnose anxiety. It is a frequently used, highly effective, and clinically validated assessment tool for anxiety disorder symptoms [28]. GAD-7 offers a psychometrically sound tool that may be used to identify patients with anxiety disorders in primary care settings as a cost-effective and simply implementable tool. Responses are on a 4-point Likert scale. The current study defined caregivers whose score was above 4 as having anxiety symptoms (GAD-7 score >4). Detailed description of the GAD-7 scale is available online [29]. In the overall sample, in SCD caregivers and HIV caregivers, the GAD-7 scale had a Cronbach alpha coefficient at 0.78, 0.78, and 0.63, respectively.

### 2.7. Patient Health Questionnaire (PHQ-9)

The 9-item Patient Health Questionnaire (PHQ-9) was used to diagnose depression. It is a commonly used instrument in primary care as it can offer practitioners a desirable numerical and “objective” diagnosis of depression and permits gauging of its severity [30]. PHQ-9 has demonstrated strong validity and reliability as well as greater adaptability even in African settings [31, 32]. It is a quick, easy, accurate, and reliable measuring method for diagnosing depression and determining its severity. Respondents indicate frequency of depression symptoms in the preceding 2 weeks on a 4-point scale. The current study defined caregivers whose score was above 4 as having depressive symptoms (PHQ‐9 score >4). Detailed description of the PHQ-9 scale is available online [33]. In the overall sample, in SCD caregivers and HIV caregivers, the PHQ-9 scale had a Cronbach alpha coefficient at 0.79, 0.78, and 0.58, respectively.

For both GAD-7 and PHQ-9, any participant with a score above 14 received immediate psychological consultation prior to leaving the health facility. Similarly, immediate psychological consultation was offered to every participant who had a PedQL FIM score below 30 [34].

### 2.8. Data Collection Procedure

Cases were recruited in a successive and convenient manner on alternate days at the SCD and HIV centers. During the regular health talks at the two centers, a 10-minute time slot was allocated each day to clarify the purpose and objectives of the study. Initially, the researchers communicated orally to express their intentions, and interested participants then approached them individually for a detailed explanation of the study. We entertained any questions and answered them accordingly. We then requested volunteer participation in the study. Caregivers who agreed to participate were registered, and only those who fitted the selection criteria were recruited. Caregivers could decide to be interviewed immediately after consultations or later at their appointed time and day based on their schedule. This was carried out after consultations in order not to interfere with their consultation programme. Interviews were conducted using an interviewer-administered questionnaire method to facilitate question clarification and improve data quality. These interviews were conducted in a quiet, comfortable room without the patient present (except for infants <1 year old) to minimize biased responses. The average interview duration was 20 minutes.

### 2.9. Data Management and Analysis

Personally identifiable information was removed from the data, which was securely stored in an encrypted online drive with limited access, protected by passwords.

Data analysis was performed using SPSS 23.0 software. Continuous variables were normally distributed and presented as mean ± standard deviation (SD), while categorical variables were presented as numbers (percentage). The chi-square test was used to analyze differences between two or more categorical variables, while Student's *t*-test was used for differences between categorical and continuous variables. A Pearson correlation test was used to determine linear association between continuous variables and the total PedsQL FIM score. We used a multivariable linear regression model to identify factors independently associated with quality of life in these two subpopulations. Variables, which were both clinically relevant such as caregiver's age and gender, depression score, anxiety score, patient's age and gender, and estimated cost of care (drugs, hospital fees, transport, and communication) and were found to be statistically significant on univariable analysis (*p* < 0.05), were included in the multivariable analysis. Regression analysis results were presented as unstandardized coefficients (B) with corresponding 95% confidence intervals (CIs). The reliability of the scales used was assessed using Cronbach's alpha.

### 2.10. Ethical Considerations

The study obtained ethical clearance from the Institutional Review Board (IRB/UD) with the reference number 3538/IEC-UD/03/2023/T. All participants provided informed consent after receiving a detailed explanation of their rights and associated risks. The tools used in the research are freely available online for academic purposes. Data collection maintained anonymity, and interviews were conducted in a private room to ensure confidentiality. Measures were taken to address potential psychological harm, with immediate psychological care provided to caregivers in distress. Equal access to participation was ensured for all potential eligible participants, regardless of socioeconomic, racial, or demographic differences.

### 2.11. Operational Definitions

1. Caregiver: An adult aged 21 years and above who lives and is the principal care provider to at least one child with SCD or HIV, during steady state, illness, or disability.
2. Anxiety: Anxiety refers to the manifestation of worry, nervousness, or discomfort arising from the anticipation of potential harm or threat [35]. For this study, participants with a score of 5 or above on the GAD-7 scale were considered to exhibit significant levels of anxiety.
3. Depression: Depression is a mood disorder characterized by enduring feelings of sadness and a diminished interest in activities [36]. In this study, caregivers who scored 5 or higher on the PHQ-9 questionnaire were identified as experiencing significant levels of depression.
4. Quality of life: Quality of life (QoL) relates to the overall well-being and satisfaction, encompassing both positive and negative aspects, of a population or an individual at a particular moment [17]. In this study, the quality of life was measured using the score obtained from the PedsQL FIM scale.

## 3. Results

### 3.1. Participant Recruitment

**Figure 1:**
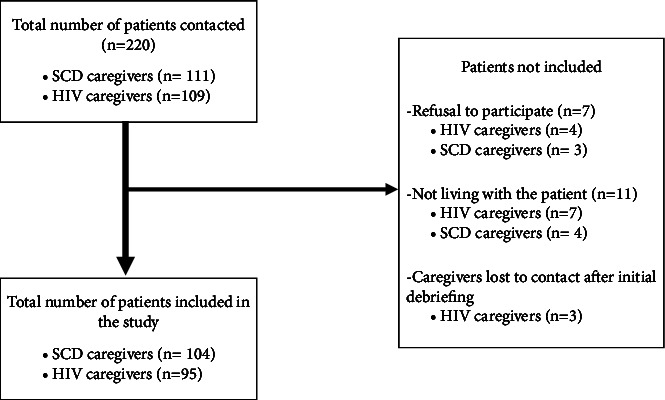
Recruitment flowchart of caregivers of SCD and HIV at Laquintinie between February and May 2023. SCD = sickle cell disease; HIV = human immunodeficiency virus.

We approached 220 participants and retained 199 participants gaining a response rate of 90.5%. The participants were composed of 104 (52.3%) caregivers of patients with SCD, and 95 (47.7%) were caregivers of participants with HIV as shown in Figure 1. There were no missing data in our sample.

### 3.2. Patients' Characteristics

The overall age range was 11 months–17 years, with a mean age of 9.73 (±4.67 SD) years. Age distribution was as follows: older children aged 6–12 years (*n* = 88, 44.22%), adolescents aged 13–17 years (*n* = 65, 32.66%), young children aged 1–5 years (*n* = 45, 22.61%), and infants aged below 1 year (*n* = 1, 0.5%). Most patients were male (*n* = 103, 51.7%), giving a male to female ratio of 1.07. Only SCD patients had been hospitalised the month prior to the interview (*n* = 24, 12.1%), as shown in Table 1.

### 3.3. Caregivers' Characteristics

The overall age range was 21–79 years, with a mean age of 40.47 ± 10.18 years. Most of the caregivers were females (*n* = 168, 84.4%), giving an M/F ratio of 1/5.4. Overall, caregivers had lived with the patient for 9.14 ± 4.75 years. The most represented ethnic group was Grassfield (*n* = 86, 43.2%), followed by the Fang–Beti (*n* = 49, 24.6%), Sawa (*n* = 45, 22.6%), and Sudano–Sahel (*n* = 19, 9.5%). Most caregivers were married (*n* = 83, 41.7%), auto-employed/independent (*n* = 81, 40.7%), and had at most secondary-level education (*n* = 119, 59.8%). Caregivers were the biological parent in 81.4% of cases (*n* = 162). Most caregivers (*n* = 85, 42,71%) had monthly revenue between 50.000 and 100.000 FCFA, closely followed by caregivers with monthly revenue below 50,000 FCFA (*n* = 79, 39.7%). Only 5.0% (*n* = 10) of the overall sample reported to spend more than 75,000 FCFA, all of whom were SCD caregivers. All caregivers of HIV patients reported spending <75,000 FCFA on medications. Relating to expenditures on hospital bills, 46.2% (*n* = 92) of the overall sample spent >75,000 FCFA, all of whom were SCD caregivers. A small proportion of patients (*n* = 7, 3.5%) reported spending more than 36,000 FCFA on transport and communication.

### 3.4. Score Components

**Figure 2:**
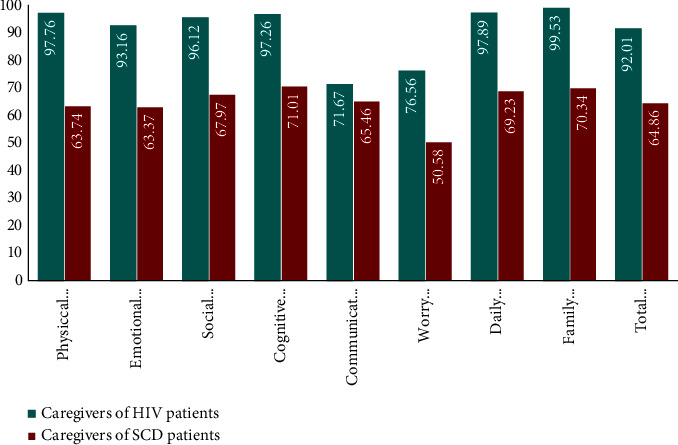
Difference in individual domain scores of the PedQL FIM scale between caregivers of HIV and SCD at Laquintinie between February and May 2023.

Each item of the PedsQL FIM score had a significant correlation with the total PedsQL FIM score (*p* value < 0.001 for each item). All individual domain scores were different between SCD and HIV caregivers as shown in Figure 2. These differences were significant (*p* < 0.001) except for the communication domain (HIV = 71.67, SCD = 65.46, *p*=0.054).

### 3.5. Anxiety, Depression and Quality of Life

Anxiety was present in 24.6% (*n* = 49) of cases: only one of the caregivers of patients with HIV had anxiety. Depression was present in 26.1% (*n* = 52) of cases: all were caregivers of patients with SCD, giving a prevalence of 50% among caregivers of patients with SCD. The mean anxiety and depression scores were 3.63 ± 4.17 and 3.29 ± 4.6, respectively. The mean quality of the life score was 77.82 ± 19.93, and caregivers of patients with HIV had a better quality of life than SCD caregivers (93.0 ± 7.35 SD versus 64.86 ± 9.20 SD, *p* < 0.001) as shown in Table 1. No HIV caregiver had depression, and only 1.05% (*n* = 1) had anxiety. Two patients reached the distress threshold on the anxiety scale only, two patients on the depression scale only, one patient on the quality of life scale only, and 7 seven patients on at least two of these sales. All these patients (*n* = 12) were caregivers of patients with SCD and were seen by the psychologist immediately.

### 3.6. Factors Associated with Quality of Life

The number of hospitalisations in the previous 12 months (*r* = −0.44, *p* < 0.001), GAD-7 score (*r* = −0.79, *p* < 0.001), PHQ-9 score (*r* = −0.8, *p* < 0.001), age of the caregiver (*r* = 0.25, *p* < 0.001), age of the patient (*r* = 0.21, *p*=0.002), number of years living with the patient (*r* = 0.16, *p*=0.028), and duration since diagnosis (*r* = 0.19, *p*=0.008) correlated significantly with quality of life. Moreover, quality of life was significantly associated with being hospitalised the previous month (*p* < 0.001), being a female caregiver (*p*=0.002), spending less than 75.000 FCFA on medications (*p* < 0.001) and hospital bills (*p* < 0.001), and having anxiety (*p* < 0.001) and depression (*p* < 0.001) as shown in Table 2. Caregivers' employment (*p*=0.061), marital status (*p*=0.069), ethnic group (*p*=0.328), and monthly revenue (*p*=0.586) were not associated with quality of life.

On multivariable analysis, PHQ-9 score (*B* = −1.52, 95% CI = [-2.08; −0.96], *p*=<0.001), GAD-7 score (*B* = −1.46, 95% CI = [-2.09; −0.83], *p*=<0.001), spending less than 75 000 FCFA on medications monthly (*B* = 12.13, 95% CI = [5.73; 18.94], *p*=<0.001), and being a SCD caregiver (*B* = −11.62, 95% CI = [-18.46; −4.78], *p*=0.001) were factors independently associated with quality of life as shown in Table 3.

## 4. Discussion

In our context, when available, psychological care services are mainly offered to patients and not caregivers. Caregivers are generally excluded despite the fact that they play a central role in providing medical and psychological care to patients and also bear all care-related expenditures [11, 37]. The purpose of our study was to characterise the quality of life (QoL) in caregivers of paediatric patients with SCD (SCDc) and compare it to caregivers of paediatric patients with HIV (HIVc) using the PedsQL FIM scale. We measured factors that could possibly influence the QoL in our population based on available literature, tested their influence on QoL, and finally compared these factors between the two subpopulations of interest.

Caregivers were predominantly females as similarly reported by other studies [1, 38, 39]. Numerous studies have reported that female caregivers are able to sacrifice their social lives, are more emotionally invested in the sick compared to male caregivers, and even when accessible, ask for less assistance from others, and therefore tend to experience depression more frequently than males [40, 41]. However, in our study, though females predominated, caregiver's gender did not influence quality of life.

Using the PHQ-9 score, one in two (50%) SCDc had depression in our sample. In the USA, using the Center for Epidemiological study—Depression Scale Revised (CESD-R-10), prevalence of depression was reported at 40% [42]. However, a context-similar study conducted in Kenya has reported an even higher prevalence of depression at 69.53% [1]. Though cycles of negative cognitions, emotions, and actions may impact coping strategies in caregivers generally, financial hardship may be a major factor contributing to the high prevalence of depression in African settings [43–46]. In our study, the prevalence of anxiety among SCD caregivers was 46.2%. It has been reported as low as 16% in the USA [42] and as high as 92.6% in Nigeria [47]. Recurring lengthy clinic appointments and increased psychological stress due to the unpredictability of sickle cell crises's outcome are factors promoting anxiety in caregivers of patients with SCD [48]. No HIV caregiver had depression, and only 1.05% had anxiety. This finding was contrary to the median PHQ-9 at 6.0 (denoting mild depression) obtained by Mwangala et al. in Kenya [49]. The poor reliability of the PHQ-9 questionnaire in HIV caregivers in our study (Cronbach alpha coefficient at 0.58) may explain why these values differ. Other tools like the Beck Depression Inventory-II (BDI-II) and the Center for Epidemiology Studies Depression Scale (CES-D) may provide suitable alternatives [50], but these tools also need to be validated in large-scale studies in Cameroon.

To evaluate the quality of life of caregivers, the PedsQL FIM score was used. The mean PedsQL FIM score in SCDc was 64.86 ± 9.20. This was lower than that reported in the USA (75.7) but much higher than that reported in Kenya (43.76) [1, 42]. In our study, depression score, anxiety score, spending more than 75,000 FCFA on medications monthly, and being a SCDc had a negative impact on the quality of life of caregivers. Depression is a recognized factor influencing QoL [9, 49]. Untreated anxiety and depression could result in impaired economic productivity, reduced ability to perform work and social roles, loss of relationships, physical decline, and problem-solving deficits for the caregivers, hence negatively impacting their QoL [49]. Mwangala et al. [49] also reported that caregiver's age and secondary level of education negatively influence the QoL of caregivers, while Van den tweel [9] additionally reported that limitation in daily activities and vitality negatively influenced QoL. SCD is a chronic disease, which may lead to debilitating complications like stroke [3, 51, 52] and femoral osteonecrosis [53, 54] which could significantly impair patient's mobility and autonomy making them fully dependent on caregivers. In addition, the intensity of pain crises may cause insomnia and psychological distress in caregivers, which may affect their quality of life [55]. We reported a positive correlation between caregiver's age and quality of life; however, this was not a significant independent factor. Increasing the caregiver's age has been reported to be associated with less anxiety and depression and therefore better quality of life. This is believed to be due to better stress coping strategies with advanced age [56]. Contrary to some reports [49, 56, 57], educational status did not affect quality of life in our study. It is assumed that education influences not just a person's income but also their ability to make wiser choices about their health and family. People with higher levels of education also have much lower unemployment rates [58].

Financial barrier, which may impair easy access to healthcare services in African context, is likely to have a big influence on quality of life [59]. In our study, spending less on medications improved QoL. Cameroon has a national programme for HIV, which gives room for better organisation of patient care and free access to most, if not, all HIV-related health services. Free services promote healthcare access and enables caregivers to redirect already limited resources to other sectors of life, thereby reducing financial stress and consequently improving quality of life. Higher expenditures per hospitalisation, higher number of hospitalisations yearly [24], and the unpredictability of crises generate a more significant financial burden for SCD caregivers compared to HIV caregivers. With all these, it is therefore not surprising that HIV caregivers have a better quality of life than SCD caregivers.

The monocentric nature of the study, the use of the convenience sampling technique, the use of scales with limited reliability, and the nonevaluation of disease severity constitute methodological limitations and therefore limit the interpretation of our results. Nonetheless, this study is novel as it addresses a significant issue in the care of patients with SCD and generates baseline data for further studies. It is the first Cameroonian study that used a standardised scale to evaluate and determine factors influencing quality of life in caregivers of HIV and SCD.

## 5. Conclusion

Quality of life is lower in caregivers of patients with SCD compared to HIV. In order to improve care in children and adolescents with SCD and quality of life of their caregivers, psychological care should be geared towards preventing, diagnosing, and treating depression and anxiety in SCD caregivers. Subsidization of medication expenditures may be beneficial in improving quality of life in caregivers with SCD.

## Figures and Tables

**Table 1:** Comparing selected variables between caregivers of SCD and HIV at Laquintinie between February and May 2023.

Variables	HIVc (*n* = 95)	SCDc (*n* = 104)	Overall sample	*p* value
*Patient characteristics*				
Mean age in years	11.33 (±3.93SD)	8.28 (±4.87SD)	9.73 (±4.69SD)	**<0.001**
Mean number of hospitalisations in the past 12 months	0.24 (±0.78SD)	1.63 (±1.60SD)	0.97 (±1.45SD)	**<0.001**
Duration since disease diagnosis	8.42 (±4.13SD)	5.64 (±4.14SD)	6.97 (±4.36SD)	**<0.001**
Male gender	49 (51.58%)	54 (51.92%)	103 (51.76%)	0.961
Hospitalised in the previous month	—	24 (23.1%)	24 (12.06%)	**<0.001**

*Caregivers' characteristics*				
Mean age in years	42.27 (±10.34SD)	38.82 (±9.78SD)	40.47 (±10.18SD)	**0.016**
Mean number of years living with the patient	10.48 (±4.27SD)	7.92 (±4.84SD)	9.14 (±4.75SSD)	**<0.001**
Female gender	75 (78.95%)	93 (89.42%)	168 (84.42%)	**0.042**
Monthly revenue <50, 000 FCFA	40 (42.11%)	39 (37.5%)	79 (39.7%)	0.507
Monthly expenditures on medications <75,000 FCFA	95 (100%)	94 (90.39%)	189 (94.97%)	**0.002**
Monthly expenditures on hospital bills <75,000 FCFA	93 (97.89%)	8 (7.69%)	101 (50.75%)	**<0.001**

*Anxiety, depression, and QoL metrics*				
Mean PHQ-9 score	0.56 (±0.88SD)	5.79 (±5.18SD)	3.29 (±4.6SD)	**<0.001**
Mean GAD-7 score	1.34 (±1.27SD)	5.71 (±4.78SD)	3.63 (±4.17SD)	**<0.001**
Mean PedsQL FIM score	93.01 (±7.35SD)	64.86 (±9.20SD)	77.82 (±19.93SD)	**<0.001**

SD = standard deviation. PedsQL FIM = Paediatric Quality of Life Family Impact Module. GAD-7 = 7-item Generalised Anxiety Disorder Scale. PHQ-9: 9-item Patient Health Questionnaire Scale. Bold values represent statistical significance at *p* < 0.05.

**Table 2:** Categorical variables associated with quality of life in caregivers of SCD and HIV at Laquintinie between February and May 2023.

Variables	Mean PedsQL FIM scale score ± SD	
	Overall sample (*n* = 199)	*p* value^*∗*^
*Patient characteristics*		
Hospitalised in the previous month	60.33 (±16.26SD)	
(Ref: no hospitalisation)	80.22 ± 19.21SD	**<0.001**

*Caregivers' characteristics*		
Female gender	75.95 (±20.62SD)	
(REF: male gender)	87.97 ± 11.31SD	**0.002**
Monthly expenditures on medications <75,000 FCFA	79.2 (±19.18SD)	
(REF: medication >75,000 FCFA)	51.81 ± 16.22SD	**<0.001**
Monthly expenditures on hospital bills <75,000 FCFA	90.26 (±10.66SD)	
(REF: hospital bills >75,000 FCFA)	65.0 (±19.5SD)	**<0.001**

*Anxiety and depression metrics*		
Depression present	52.52 (±16.35SD)	**<0.001**
(REF: depression absent)	86.77 (±11.69SD)	
Anxiety present	51.08 (±15.62SD)	
(REF: anxiety absent)	86.56 (±11.72SD)	**<0.001**

REF = reference category. SD = standard deviation. PedsQL FIM = Paediatric Quality of Life Family Impact Module. NaN = not applicable since single patient. *p* values^*∗*^ represent comparative analysis of the REF category and the exposure category in the overall sample.

**Table 3:** Univariate and multivariable analysis of factors associated with quality of life in caregivers of SCD and HIV consulted at Laquintinie between February and May 2023.

Variables included in the model	Univariate analysis model				Multivariable analysis model			
	B	95% confidence interval for B		*p* value	B	95% confidence interval for B		*p* value
		Lower bound	Upper bound			Lower bound	Upper bound	
Age of the caregiver	0.5	0.23	0.76	**<0.001**	0.12	-0.04	0.28	0.152
Female caregiver (REF = male gender)	−12.02	−19.58	−4.47	**0.002**	−1.14	−5.07	2.8	0.568
Number of years living with the patient	0.65	0.07	1.23	**0.028**	−0.02	−0.65	0.61	0.945
Age of the patient	0.91	0.32	1.5	**0.002**	−0.21	−0.97	0.54	0.575
Duration since disease diagnosis	0.86	0.22	1.49	**0.008**	0	−0.51	0.51	0.994
Number of hospitalisations in the previous year	−6.05	−7.79	−4.31	**<0.001**	−0.27	−1.5	0.96	0.663
Mean PHQ-9 total score	−3.46	−3.83	−3.09	**<0.001**	−1.52	−2.08	−0.96	**<0.001**
Mean GAD-7 total score	−3.76	−4.17	−3.34	**<0.001**	−1.46	−2.09	−0.83	**<0.001**
Caregivers' group: SCDc (REF: HIVc)	−27.15	−31.26	−23.05	**<0.001**	−11.62	−18.46	−4.78	**0.001**
Hospitalised during the previous month: yes. (REF = no)	−19.89	−28.04	−11.74	**<0.001**	1.6	−3.29	6.49	0.517
Spending less than 75k on medications (REF: spending 36k or more on medications)	27.39	15.14	39.64	**<0.001**	12.33	5.73	18.94	**<0.001**
Spending less than 75k on hospitalisation (REF: spending 75k or more on medications)	25.26	20.92	29.59	**<0.001**	0.11	−6.39	6.61	0.972

B: unstandardised coefficient. Bold values represent statistically significant variables representing a two-tailed *p* value at <0.05. SCDc = caregivers of patients with sickle cell disease. HIVc: caregivers of patients with HIV.

## Data Availability

The data that support the findings of this study are available from the corresponding author upon reasonable request.

## Additional Points

*What Is Known on the Topic?* (i) Paediatric chronic illnesses like HIV and SCD place a significant burden on children and their families that may manifest as symptoms of depression and anxiety. (ii) This impact on families is worse in low- and middle-income countries (LMICs) due to inadequate social welfare and healthcare services. (iii) In Cameroon, however, only HIV benefits from a longstanding free-service national programme, which considerably alleviates burden of care. *What This Study Adds?* (i) One in two SCD caregivers has anxiety and depression. Comparatively, no HIV caregivers have depression, and only 1.05% (1/95) have anxiety. (ii) Quality of life is lower in caregivers of patients with SCD compared to HIV. (iii) Preventing depression and anxiety as well as advocating for the subsidization of medications may improve quality of life in SCD caregivers.
